# Systemic Administration of Avian Defensin 7: Distribution, Cellular Target, and Antibacterial Potential in Mice

**DOI:** 10.3389/fmicb.2019.00541

**Published:** 2019-03-26

**Authors:** Geoffrey Bailleul, Rodrigo Guabiraba, Isabelle Virlogeux-Payant, Isabelle Lantier, Jérôme Trotereau, Florence B. Gilbert, Agnès Wiedemann, Angélina Trotereau, Philippe Velge, Catherine Schouler, Anne-Christine Lalmanach

**Affiliations:** ISP, INRA, Université de Tours, UMR 1282 Centre INRA Val de Loire, Nouzilly, France

**Keywords:** chicken, defensins, mouse, macrophages, *Salmonella* Typhimurium

## Abstract

Defensins are natural antimicrobial peptides. The avian beta-defensin AvBD7 isolated from the chicken bone marrow possess broad antibacterial spectrum and strong resistance to proteolysis. However, its ability to fight systemic infections of major concern for public health, such as salmonellosis, is unknown. As a first approach, fluorescence labeling of AvBD7 allowed to track its systemic distribution after intraperitoneal injection in mice using whole body live imaging. It was associated to peritoneal cells and to deeper organs such as the liver. In the next step, the use of labeled AvBD7 allowed to observe its interaction with murine macrophages in culture. After incubation, it was able to penetrate inside the cells through an endocytosis-like mechanism. Furthermore, natural AvBD7 contributed to the control of intracellular multiplication of a multidrug resistant *Salmonella* strain, after incubation with infected macrophages. Finally, administration in a model of systemic lethal *Salmonella* infection in mice led to significant improvement of mouse survival, consistently with significant reduction of the liver bacterial load. In conclusion, the results reveal a hitherto unknown intracellular antibacterial effect of AvBD7 in *Salmonella* target cells and support AvBD7 as a candidate of interest for the treatment of infectious diseases caused by multidrug-resistant pathogenic *Enterobacteriaceae.*

## Introduction

Host defense peptides are important antimicrobial components of animal’s innate immunity. Defensins represent the most abundant and the most conserved family of these cationic peptides throughout evolution (Ganz, 2003). In the past decades, they have been described not only able to kill a broad variety of microorganisms, mainly bacteria and fungi, but also able to modulate the host immune response mainly in mammals (Semple and Dorin, 2012; Hancock et al., 2016). The direct antimicrobial activity of defensins has been attributed to a mechanism of bacterial membrane disruption without specific molecular target (Taylor et al., 2008). In many species of gram-negative bacteria, the charge on the outer membrane is modulated by the two-component system PhoPQ regulon affecting cationic antimicrobial peptide sensitivity through modulation of the PmrA regulon, which controls a set of genes that mediate decoration of the outer membrane with the positively charged moieties ethanolamine and 4-aminoarabinose (Gunn et al., 2000). Bacterial resistance to cationic antimicrobial peptides is nevertheless clearly more difficult to attain than for conventional antibiotics through mutation of the specific target, and it can be considered as a “costly” solution for the bacteria to survive (Hancock and Sahl, 2006). Moreover, the cross-resistance of laboratory-selected mutants to other peptides seems to be limited (Samuelsen et al., 2005), and the importance of immunomodulatory properties of these peptides, which would not be affected by antimicrobial resistance, has been increasingly recognized (Hancock et al., 2016). Taken together, these properties place defensins as a potential therapeutic solution alternative or complementary to conventional antibiotics. However, in spite of an abundant database of antimicrobial peptides^1^, only a few of them have reached phase II–III clinical trials and mainly for topical applications (Mahlapuu et al., 2016). This can be due to a low stability of the peptides under physiopathological conditions and/or to their weak systemic bio-distribution in the case of infections targeting remote organs.

Chickens possess 14 avian beta-defensins (AvBDs) and no alpha-defensins (van Dijk et al., 2008; Cuperus et al., 2013). AvBDs are characterized by cationicity and structure with the typical beta-sheet stabilized by three disulfide bonds between six highly conserved cysteines while primary sequences are diverse (Derache et al., 2012). Among these avian defensins, AvBD1 and AvBD2 can be isolated together with AvBD7 from chicken bone marrow, and all exhibit broad antimicrobial spectrum toward Gram-negative and Gram-positive bacteria, with micromolar range of minimum inhibitory concentrations (MICs) (Derache et al., 2009). It is worth to note AvBD2 and AvBD7 as major antimicrobial components of chicken intestinal epithelium and granulocytes produced by the bone marrow and infiltrating infected tissues. Importantly, there is a strong resistance of AvBD7 to degradation under proteolytic conditions, as compared to AvBD2 (Bailleul et al., 2016). Nine serine and cysteine proteases incubated with AvBD7 do not impair its antimicrobial activity toward gram-negative *Enterobacteriaceae*. This resistance to proteolysis is associated to the highly stabilized structure of AvBD7, with the N-terminal extremity being (1) protected by a first pyroglutamate residue, (2) stabilized by a salt bridge between D9 and R12 residues, and (3) covering the C-terminal part, thus limiting access to proteases with amino- and/or carboxy-peptidase activity. This remarkable property appears to be very characteristic of AvBD7 since other defensins such as HBD2 and HBD3 can be inactivated by proteases (Taggart et al., 2003). Resistance to proteolytic conditions is of major importance during infection when proteases increase within the inflammatory foci (Rodenburg et al., 2007; Augustin et al., 2013). Therefore, this peculiar stability of AvBD7 could be a major advantage *in vivo* to fight bacterial infections.

*Salmonella* is a gram-negative bacterium causing important infectious diseases with major impact on public health worldwide. It manifests either as a systemic disease such as the one caused by *Salmonella enterica* serovar (*S*.) Typhi in humans and commonly named typhoid fever (Mogasale et al., 2014) and by Typhimurium in mice, or as an enteric disease such as the one caused mainly by *S.* Enteritidis or *S*. Typhimurium in humans^2^. *Salmonella* is a facultative intracellular bacterium able to survive and multiply within macrophages, that are an abundant leukocyte population in the peritoneal cavity (Murch et al., 1984), unless these cells become activated to exert antimicrobial functions (Dougan et al., 2011). The emergence of resistance to antibiotics in *Salmonella* strains is a serious health problem worldwide (Lammie and Hughes, 2016). The number of strains developing multidrug resistance (MDR) phenotype has increased in many countries since the first emergence of MDR *S*. Typhimurium DT104 strain in 1990 (Crump et al., 2015).

There is a lack of knowledge on the ability of defensins to exert antimicrobial activity toward MDR bacterial strains. However, it is interesting to notice that AvBD7 exhibits stronger antibacterial activity against *Salmonella* spp. as compared to AvBD2 (Derache et al., 2009) or to AvBD103b (Thouzeau et al., 2003), suggesting AvBD7 could be used in the treatment of salmonellosis. There are several mouse salmonellosis models available to assess vaccine and antimicrobials efficacy (Higginson et al., 2016). These range from models of colonization or lethality, through infection by the oral, intravenous, sub-cutaneous or intraperitoneal route, to those involving complex surgical techniques. Of these various routes, oral inoculation with *Salmonella* most closely resembles the natural route of infection in humans. However, the intraperitoneal (i.p.) route has often been used as it gives a very robust model that results in patterns of infection of the reticulo-endothelial system and death similar to that observed after oral inoculation of mouse (Santos et al., 2001). Furthermore, it offers the advantage of reducing inter-animal variability, by avoiding particularly individual variations of gastro-intestinal physiological conditions and flow. Although the oral route of infection is pertinent to highlight the role played by an endogenous antimicrobial peptide during enteric salmonellosis, as elegantly demonstrated for a human alpha-defensin (HD5) by Salzman et al. (2003), our study rather seeks to estimate whether an exogenous antimicrobial peptide could tackle the devastating systemic disease. Using an intraperitoneal mouse model of salmonellosis, Maiti and colleagues revealed that two human beta-defensins (HBD1 and HBD2) are efficient in attenuating the disease and the bacterial load (Maiti et al., 2014). In this context, we aimed to gain initial insights on whether systemic administration of AvBD7 in mice would lead to exploitable bio-distribution and to beneficial effects on host-infected cells, organs, and on host survival during lethal systemic salmonellosis. Our main interest is to highlight a potential therapeutic usage of AvBD7 as an alternative to conventional antibiotics in mammals.

## Materials and Methods

### Avian Defensin, Bacterial Strains, Cell Line, and Animals

Twenty ROSS PM3 chickens aged 6–9 weeks provided by PEAT UE 1295 (Centre INRA Val de Loire, France) were sacrificed at the PFIE UE 1277 animal facility (Centre INRA Val de Loire, France) by electro-narcosis and cervical dislocation, in order to collect femurs and tibias post mortem. This was performed in strict compliance with European Union Directive 2010/63/EU for animal killing and under authorization and supervision of official veterinary services (agreement number C-37-175-3 delivered to the PFIE animal facility by the veterinary service of the Département d’Indre et Loire, France). In addition, the procedure was performed in strict compliance with legal dispositions applicable in France, mentioning animal euthanasia with only purpose of organ or tissue use is not considered as an experimental procedure and thus not under obligation of submission to ethics committee for approval (Ordinance 2013-118, article R.214-89, published in the Journal Officiel de la République Française # 0032 of the February 7, 2013, pp. 2199). Avian beta-defensin AvBD7 was extracted and purified from the bone marrow of collected femurs and tibias, as previously described (Derache et al., 2009; Bailleul et al., 2016).

*Salmonella* strains were selected according to MDR resistance phenotype: *S.* Kentucky ST198-X1 02-8141 (Doublet et al., 2008) and *S.* Typhimurium DT104 BN10055 (Baucheron et al., 2004), kindly provided by Dr. Benoît Doublet (ISP UMR 1282, Centre INRA Val de Loire, France). The use of a frozen stock of bacterial strains allowed preparation of inoculum for either *in vivo* or *in vitro* studies. Counting CFU after plating serial dilutions of the bacterial suspension on tryptic soy agar (TSA) allowed bacterial quantification. Radial diffusion assay was used to measure the antimicrobial activity of AvBD7 toward each of the *Salmonella* strains by MIC determination as previously described (Derache et al., 2009). In order to obtain fluorescent bacteria for cellular analyses by confocal microscopy, *Salmonella* strains harboring pFPV-mCherry/2 were prepared by electroporation, as previously described (Drecktrah et al., 2008).

Mouse macrophage cell line RAW 264.7 (ATCC^®^ Number: TIB-71^™^) was cultured in Dulbecco’s Minimal Essential Medium-F12 (DMEM-F12, Gibco) complete medium containing 15 mM HEPES buffer, 2 mM glutamine, 10% FCS (Lonza), and 1% of antibiotics mixture (10,000 U/ml of PEN and 10 mg/ml of STR, Sigma-Aldrich) at 37°C under 5% CO_2_ and humidity.

Six-week-old female BALB/c mice with 20–25 g of body weight (Janvier Labs, France) have allowed *in vivo* studies. All animal experiments were performed in compliance with French and European ethical guidelines respecting the “Replacement, Reduction and Refinement” rule as much as possible and were realized at the animal facility PFIE UE 1277 (Centre INRA Val de Loire, France). The murine model of systemic salmonellosis (described in the sub-section Application to a Model of Systemic Salmonellosis below) was chosen according to Maiti et al. (2014) in spite of many animal models described (Higginson et al., 2016), in order to compare the effect of AvBD7 administration to the one obtained by Maiti and colleagues using human beta-defensins. Experimental procedure has been approved (authorization number 03749.03 delivered the 30 April 2015) by the French national ethics committee for animal experimentation (committee number 19, CEEA Val de Loire).

### Whole Body *in vivo* Imaging

For imaging of live animals, it was chosen to avoid labeling of AvBD7 on thiol groups of cysteines in order to maintain disulfide bridges (thus protein folding) which have been previously demonstrated as important in the resistance of AvBD7 to proteolytic conditions encountered *in vivo* (Bailleul et al., 2016). Even if the AvBD7 sequence does not contain reactive amine group (lysine side chains), a probe containing succinimidyl ester group can react with its hydroxyl groups (serine, threonine, and tyrosine side chains) (Madler et al., 2009). AvBD7 was thus labeled with the XenoLight CF^™^ 750 (succinimidyl ester) probe (Perkin Elmer) following the manufacturer’s recommendations, and the free dye was removed from the labeled molecule by ultrafiltration using Amicon^®^ Ultracel^®^ (0.5 ml) system with a 3 kDa cut-off (Merck Millipore). The degree of labeling was determined according to absorbance measurements at 280 and 750 nm and was of 0.15, according to the manufacturer’s recommendations. Mice were injected by i.p. route with 150 μl of sterile endotoxin-free H_2_O containing (*n* = 3) or not (*n* = 2) 100 μg of labeled AvBD7, or containing (*n* = 3) the same amount of free dye (according to absorbance measurement at 750 nm). The *In Vivo* Imaging System (IVIS Spectrum, Perkin Elmer) with auto exposure epi-illumination setting (745 nm excitation/800 nm emission) used on anesthetized animal at the indicated time points allowed to record images, according to the manufacturer’s recommendations. Living Image 4.5.2 software (Perkin Elmer) allowed performing apparatus set up, data acquisition, and data analysis. A sacrificed animal at each indicated time point allowed to measure fluorescence from collected cells and organs. Average radiant efficiency has given values of fluorescence intensity.

### Interaction With Mouse Macrophage Cell Line

In order to assess a hypothetical cytotoxicity, AvBD7 was added for 6 h at the concentration of 5 or 30 μM to RAW 264.7 cells. After scraping in ice-cold PBS, the cells were centrifuged and stained at 4°C with annexin V-FITC (BD Biosciences) and 7-aminoactinomycin D (7AAD, BD Biosciences), following the manufacturer’s instructions. After two washes, flow cytometry using a FACS Calibur machine (BD Biosciences) allowed analysis of the cells. Measuring percentages of early apoptotic cells (annexin V-FITC positive and 7AAD negative staining), of late apoptotic cells (annexin V-FITC and 7AAD double positive staining), and of necrotic cells (annexin V-FITC negative and 7AAD positive staining) after incubation with or without AvBD7 allowed to assess cell death phenomena.

In order to assess interaction of AvBD7 with macrophages, purified AvBD7 was labeled with FluoProbes^®^488-maleimide (Interchim) following the manufacturer’s instructions. Adding 1.10^5^ cells/well in 500 μl of respective complete medium overnight at 37°C allowed obtaining adherent cells on round-shaped glass slides (14 mm diameter) in a 24-well culture plate (Nunc). After rinsing the cells five times with PBS, they were incubated for 1 h with labeled AvBD7 at 3 μM in complete medium. The presence of inhibitory conditions allowed to evaluate hypothetical mechanisms of interaction of labeled AvBD7 with the cells, such as incubation at 4°C or with nystatin (50 μM, Sigma-Aldrich) to reduce membrane fluidity (Perry and Wobus, 2010), or incubation with cytochalasin D (30 μM, Sigma-Aldrich) to block actin polymerization (Miller et al., 2004) or with chlorpromazine (30 μM, Sigma-Aldrich) to block clathrin-mediated endocytosis (Pereira et al., 2014). Cells were then fixed with 500 μl of paraformaldehyde 4% (Santa Cruz Biotech) for 30 min. Nuclei were stained with DAPI (1/2000 dilution in PBS, Sigma-Aldrich) and actin was stained with rhodamine-phalloidin (1/200 dilution in PBS, Sigma) for 20 min. After mounting round-shaped slides on classical slides using Permafluor^®^ (ThermoFisher) following the manufacturer’s instructions, a Leica SP8 confocal microscope (Leica Microsystem) allowed to observe labeled cells on slides.

In order to assess interaction of AvBD7 with macrophages infected by *Salmonella*, adherent cells on round-shaped slides were inoculated with the fluorescent bacterial strain at a multiplicity of infection (MOI) of 10 for 1 h. Following gentamicin treatment at 100 μg/ml for 1 h to kill extracellular bacteria, fluorescent AvBD7 was added for 1 h to examine its interaction with infected cells by confocal microscopy. In order to evaluate AvBD7 antibacterial effect, infected cells treated by gentamicin and maintained in culture with 10 μg/ml of gentamicin and with 0, 3, or 30 μM of natural AvBD7 for 24 h were lysed with sterile water for 30 min at 4°C. Serial dilutions and plating on TSA allowed to determine CFU counts for numeration of intracellular bacteria. Percentage of growth was calculated by the ratio n24:n0 where n24 is the number of intracellular bacteria at *t* = 24 h and n0 is the number of intracellular bacteria at *t* = 0, considering as 100% the ratio obtained under control condition (untreated cells).

### Application to a Model of Systemic Salmonellosis

Ten mice were inoculated in a first experiment by intraperitoneal (i.p.) route with 140 CFU of either of the two MDR *Salmonella* strains suspended in 100 μl of sterile NaCl 0.9% solution, in order to select the appropriate strain inducing lethal salmonellosis. In a second experiment, 10 mice infected with 140 CFU of the specified *Salmonella* strain received two i.p. injections each of 100 μl containing 100 μg of AvBD7, with a 2-h interval. Control group of 10 infected mice received two injections of 100 μl of sterile endotoxin-free H_2_O. For each of the experiments, monitoring of animals twice a day during 8 days allowed recording clinical symptoms (prostration, low mobility, closed eyes, and ruffled hair) and mortality rates. In a third experiment designed for kinetics analysis of bacterial load, 30 infected and treated mice or 30 infected and mock-treated mice in the same conditions as in the second experiment were sacrificed (10 mice per time point) sequentially at 3, 6, or 24 h post-inoculation (p.i.). In parallel and for cellular controls at 6 and 24 h, 20 non-infected mice were treated or not by AvBD7. Injecting 7 ml of ice-cold PBS (Gibco) allowed peritoneal lavage by gentle massage and collection of 5 ml of the peritoneal exudate separated in two tubes, half for bacterial load analysis and half for peritoneal cells analysis. Spleen and liver collected aseptically were weighted and homogenized in 1 and 3 ml of tryptic soy broth (TSB) medium, respectively. Serial dilutions of organ homogenates and half-peritoneal exudate plated on TSA allowed counting CFU. Cells of the second half-peritoneal exudate were pelleted (1,500 g for 5 min at 4°C), re-suspended at the concentration of 5 × 10^4^ cells in 300 μl of ice-cold PBS and deposited on glass slides by centrifugation at 700 rpm for 5 min, following the manufacturer’s instructions (Shandon Cytospin 3 Centrifuge, Rankin Biomedical). Cells staining using the Diff-Quick method following the manufacturer’s instructions (Siemens) allowed differential counting of stained cells (monocytes/macrophages, basophils, neutrophils, and eosinophils) performed twice from 200 cells per slide. The mean number of cells obtained for each population was adjusted to correspond in proportion to the total number of collected peritoneal cells.

### Statistical Analysis

One-way analysis of variance followed by Tukey’s test allowed statistical comparison of gated cell percentages obtained by flow cytometry analysis between the tested conditions. Mann-Whitney U test allowed statistical comparison of bacterial load or of peritoneal cell number between different conditions at indicated time-points. Log-rank test allowed statistical comparison of mice survival curves between groups.

## Results

### Whole Body Imaging of Fluorescent AvBD7

**Figure 1:**
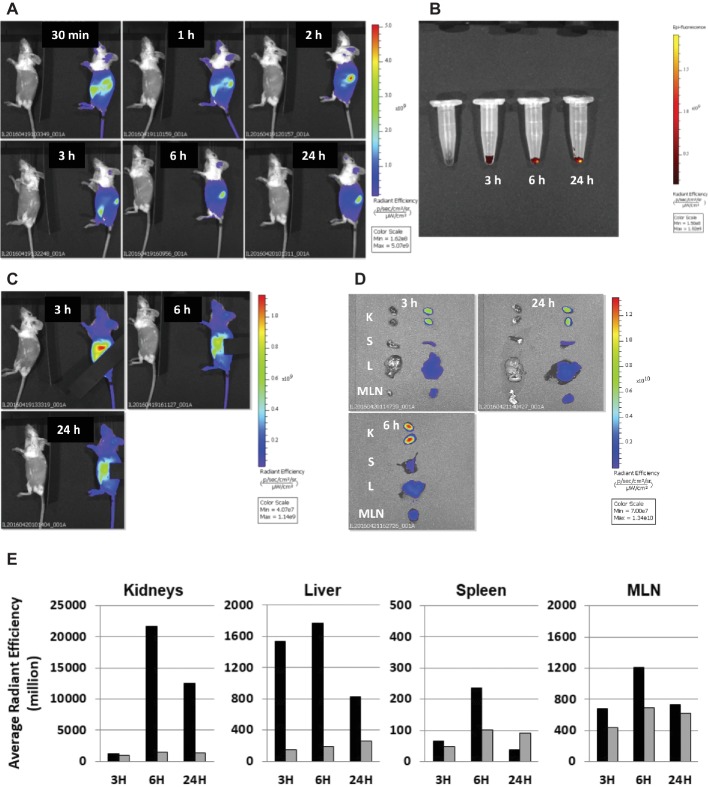
Whole body fluorescence imaging of AvBD7 *in vivo* and *ex vivo* post intraperitoneal injection in mice. Mice were injected with 100 μl of either H_2_O alone (negative control, left part of each image) or containing 100 μg of fluorescence-labeled AvBD7 or containing the same amount of free dye (according to absorbance measurement at 750 nm). Times for image acquisition are indicated on each image. **(A)** Mice imaged on left lateral view. **(B)** Pelleted peritoneal cells collected from control (left) or fluorescent AvBD7-injected mice. **(C)** Mice imaged on left lateral view, hiding the kidneys area. **(D)** Organs imaged *ex vivo* (K, kidneys; S, spleen; L, liver; MLN, mesenteric lymph node). **(E)** Quantification of AvBD7 fluorescence (black bars) by comparison to that of the free dye fluorescence (grey bars) in organs collected post i.p. injection in a sacrificed animal at three indicated times (in h). Values are given in million average radiant efficiency.

Figure 1 shows the dissemination of AvBD7 from the mouse peritoneal cavity investigated by imaging kinetics of fluorescence-labeled AvBD7 distribution over time following i.p. administration of 100 μg and by quantifying fluorescence in organs *ex vivo*. Fluorescence imaging of labeled AvBD7 in mice during the first 24 h post i.p. injection revealed early and highly positive signals in both ventral and dorsal abdominal areas, which appeared to correspond to bladder and kidney areas, respectively (Figure 1A). In addition, urine collected from labeled AvBD7-injected mice was highly positive during the first few hours postinjection (data not shown). The early detection of fluorescent signals in highly vascularized regions such as the tail and the ears was indicative of the presence of labeled AvBD7 in the bloodstream. When collecting peritoneal exudate, pelleted cells obtained were fluorescent over 24 h (Figure 1B). In order to reduce the *in vivo* fluorescence signal from the kidneys and from the bladder area, the use of a mask positioned on these areas of the abdomen allowed the detection of labeled AvBD7 from the upper ventral part of the abdomen, which could correspond to liver and spleen areas (Figure 1C). Abdominal organs removed from sacrificed mice and imaged for fluorescence detection (Figure 1D) and quantification (Figure 1E) validated this hypothesis. While the digestive tract was completely negative (data not shown), the most intense fluorescent signal was observed for the kidneys, followed in intensity by the liver and mesenteric lymph nodes, and finally, the lowest fluorescent organ was the spleen. Each organ reached the maximum fluorescence intensity at 6 h postinjection.

### Interactions of AvBD7 With Macrophages

#### AvBD7 Effect on Cell Viability

**Figure 2:**
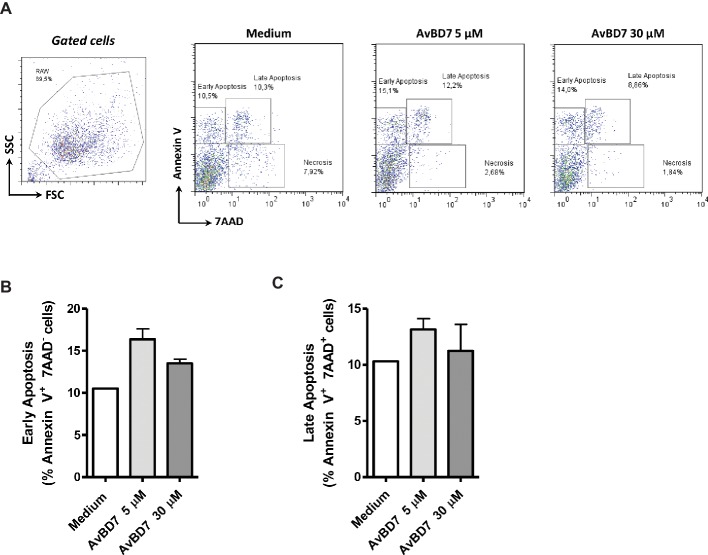
Viability of murine macrophages following exposure to AvBD7. The RAW 264.7 cells were incubated with AvBD7 at 5 or 30 μM or with medium alone for 6 h, and were stained with annexin V-FITC or 7AAD. Parameters to measure cell death were early apoptosis (annexin V-FITC positive, 7AAD negative staining), late apoptosis (annexin V-FITC and 7AAD double positive staining) and necrosis (annexin V-FITC negative and 7AAD positive staining). **(A)** Flow cytometry dot plots showing the total gated cells (left plot) and the staining of the cells in different incubation conditions indicated above each of the right plots. Cells undergoing early apoptosis (top left), late apoptosis (top right) or necrosis (bottom right) are delineated on each plot. **(B)** and **(C)** Bar graphs showing the percentages of early **(B)** and late **(C)** apoptotic cells following incubation in conditions indicated below each bar (*n* = 3).

Macrophages being a dominant cell population of the mouse peritoneal cavity, the direct effect of AvBD7 on the viability of murine macrophages was evaluated using flow cytometry analysis (Figure 2). More than 80% of the cells were viable (7AAD negative staining) after 6 h of incubation with or without AvBD7 at 5 or 30 μM concentrations (Figure 2A). There were no significant differences of percentage of cells between control (medium) group and treated (AvBD7) groups for early (Figure 2B) and late (Figure 2C) apoptosis.

**Figure 3:**
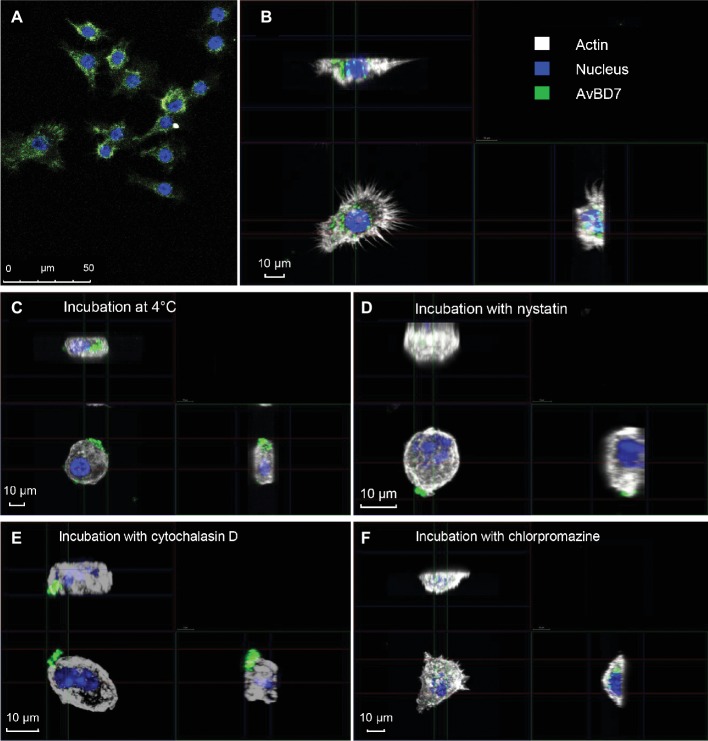
Confocal microscopy imaging of AvBD7 interaction with mouse macrophages. RAW 264.7 macrophages were incubated with 3 μM of fluorescence-labelled AvBD7 **(A)**, and **(B)**, in the presence of cytochalasin D **(C)** or of nystatin **(D)** or of cytochalasin D **(E)** or of chlorpromazine **(F)**, and were fixed and processed for immunofluorescence. Nucleus is in blue, actin in white and fluorescence-labeled AvBD7 in green. Three dimension confocal microscopy images **(B–F)** shows horizontal (bottom left) and vertical (top left and bottom right) sections of a representative cell.

In order to determine AvBD7 localization after interaction with mouse macrophages, confocal microscopy allowed to observe the cells incubated with fluorescence-labeled defensin (Figure 3). Most of the cells appeared with fluorescent defensin after 1-h incubation (Figure 3A). Three-dimensional analysis of a representative AvBD7-positive cell also labeled for actin revealed the penetration of the defensin inside macrophages, as observed by the accumulation of fluorescent AvBD7 in the cytoplasm (Figure 3B). Furthermore, to gain indications on the mechanism of entry of AvBD7 inside macrophages, the interaction experiment was performed using different inhibitory conditions. To reduce cell membrane fluidity, incubation at 4°C (Figure 3C) or with nystatin (Figure 3D) both inhibited AvBD7 intracellular translocation. In the presence of cytochalasin D inhibiting actin polymerization, it was also noticed a disrupted intracellular localization of labeled AvBD7 (Figure 3E), whereas chlorpromazine used as inhibitor of clathrin-mediated endocytosis did not block AvBD7 entry inside the cells (Figure 3F).

#### AvBD7 Effect on Infected Cells

**Figure 4:**
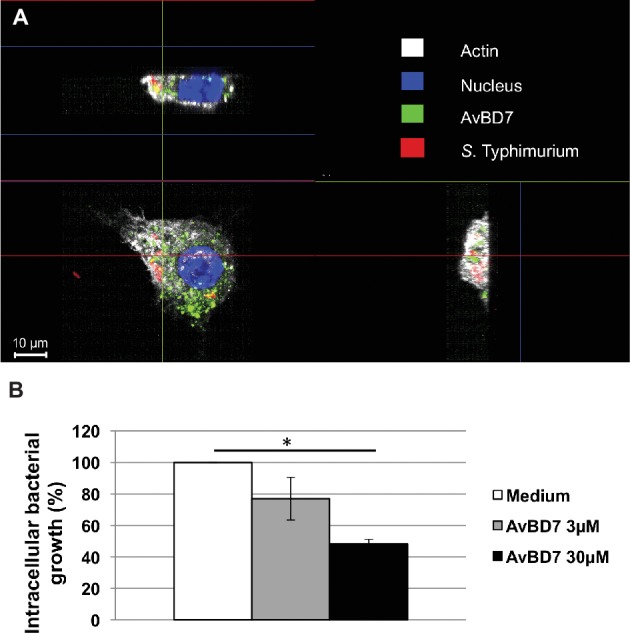
Interaction of AvBD7 with murine macrophages infected by *S.* Typhimurium. 3D confocal microscopy analysis gave the representative image **(A)** of RAW 264.7 macrophage infected by *S.* Typhimurium DT104 strain (red) and incubated with labeled AvBD7 (green). The image shows horizontal (bottom left) and vertical (top left and bottom right) sections of the cell processed for immunofluorescence. Nucleus is in blue and actin in white. Intracellular multiplication of bacteria **(B)** in RAW 264.7 macrophages infected by *S.* Typhimurium and treated or not by AvBD7. Cells were incubated with bacteria for 1 h at MOI of 10, treated with gentamicin (100 μg/ml) for 1 h, and incubated for 24 h in the absence (medium; white bar) or presence of 3 μM (grey bar) or 30 μM (black bar) of AvBD7. Results are expressed as mean percent ± standard error of the mean (each well/condition in triplicate; *n* = 3); Asterisk indicates significant difference with the non-treated cells (*p =* 0.02).

Next, the ability of AvBD7 to interact with infected cells and to control infection was investigated (Figure 4). Confocal microscopy image of a representative murine macrophage infected by *S.* Typhimurium (in red) and incubated for 1 h with fluorescence-labeled AvBD7 (in green) showed intracellular accumulation of the defensin (Figure 4A), as mentioned above. AvBD7 and bacteria inside the cell co-localized at few points observed in yellow. Furthermore, AvBD7 ability to control *Salmonella* infection inside host cells was examined. As shown in Figure 4B, incubation of infected cells for 24 h with AvBD7 (30 μM) significantly reduced by 50% the intracellular multiplication of *S.* Typhimurium in macrophages (*p* = 0.02), as compared to control condition (medium).

### Effect of AvBD7 Administration in a Mouse Model of Systemic Lethal Salmonellosis

#### Selection of the *Salmonella* Strain

Two different strains of *Salmonella* identified by their multidrug resistance phenotype were tested for their susceptibility to AvBD7 and for their ability to induce systemic lethal salmonellosis in mice (Table 1). The *S.* Typhimurium strain was the only one to induce 100% mortality at the dose of 140 CFU/animal 7 days after i.p. inoculation (Table 1). The antibacterial activity of AvBD7 measured on this *Salmonella* strain showed a MIC of 3.5 μM (19.0 mg/L). The *S.* Typhimurium DT104 strain thus susceptible to AvBD7 was appropriate for measuring the effect of AvBD7 administration *in vivo* on the systemic lethal salmonellosis in mice.

**Table 1:** Phenotypic characteristics of *Salmonella* strains.

*Salmonella* strain	Resistance to antibiotics	MIC of AvBD7^a^	Lethality in mice^b^
*Salmonella* Kentucky, ST198-X1, 02-8141	AMP, CHL, GEN, STR, SPT, TET, NAL	2.5	0
*Salmonella* Typhimurium, DT104, BN10055	AMP, CHL, STR, SPT, TET, NAL	3.5	100

a Minimum inhibitory concentrations (MICs) of AvBD7 are given in μM and were calculated using the radial diffusion assay, as previously described (Derache et al., 2009).

b Lethality in mice is given in percent and was recorded at day 7 post i.p. inoculation of 140 CFU/animal (*n* = 10).

#### Effect on Peritoneal Cells

**Figure 5:**
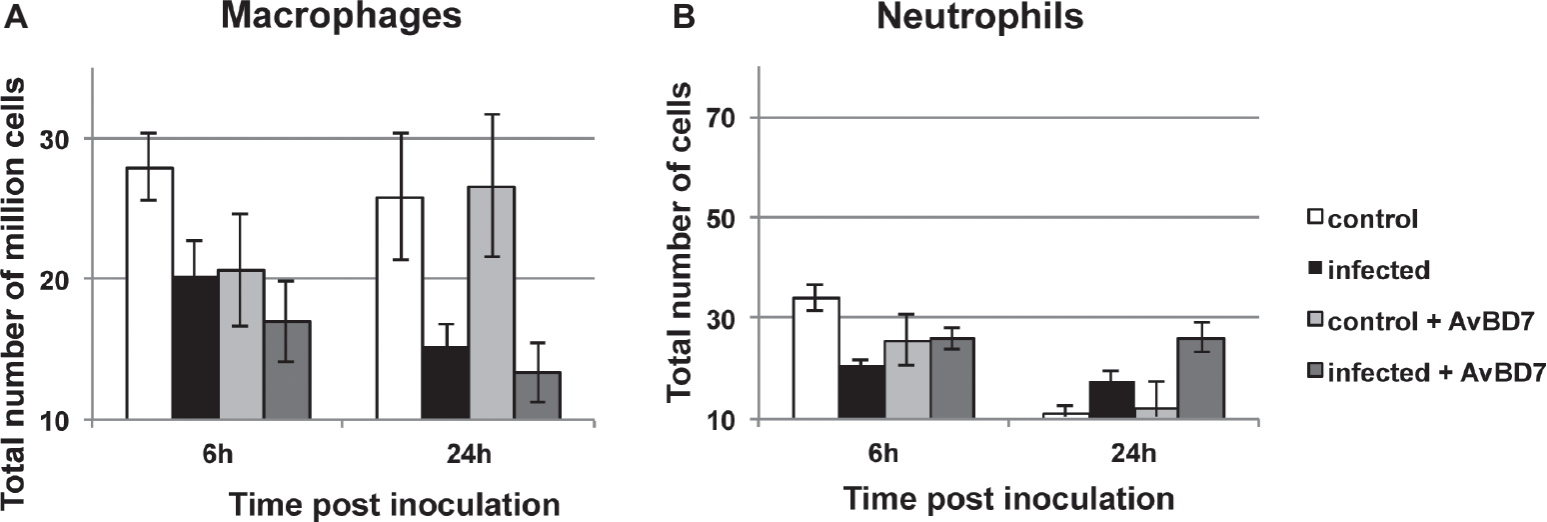
Peritoneal cell counts in mice infected or not with *S.* Typhimurium and treated or not with AvBD7, after 6 or 24 h. BALB/c mice were inoculated or not with *S.* Typhimurium and were treated or not with AvBD7, as indicated in the Materials and Methods section. Peritoneal monocytes/macrophages **(A)** or neutrophils **(B)** numbers from non-infected and non-treated animals are represented by white bars; infected and non-treated animals are represented by black bars; non-infected and treated animals are represented by light grey bars; infected and treated animals are represented by dark grey bars. Values are given as mean total number of million macrophages or as mean total number of neutrophils ± standard error of the mean (*n* = 10).

After inoculation of *S.* Typhimurium DT104 strain to induce systemic lethal salmonellosis, mice received intraperitoneal injection of 100 μl containing or not 100 μg of AvBD7, injection repeated 2 h after the first one (total 10 mg/kg of body weight). Collected peritoneal cells analyzed at 6 and 24 h p.i. allowed to define host cell types present in the peritoneal cavity and to measure their evolution in numbers during infection and/or treatment by AvBD7, as shown in Figure 5. Monocytes/macrophages represented the most abundant cell population in the peritoneal cavity of control or infected animals (Figure 5A), being 10^6^ fold more abundant than neutrophils (Figure 5B). The number of macrophages was slightly but not significantly reduced in the peritoneal cavity of mice after 24 h of infection, by comparison to non-infected mice (Figure 5A). AvBD7 injection did not significantly modify cell proportions in infected or non-infected mice.

#### Effect on Systemic Bacterial Load

**Figure 6:**
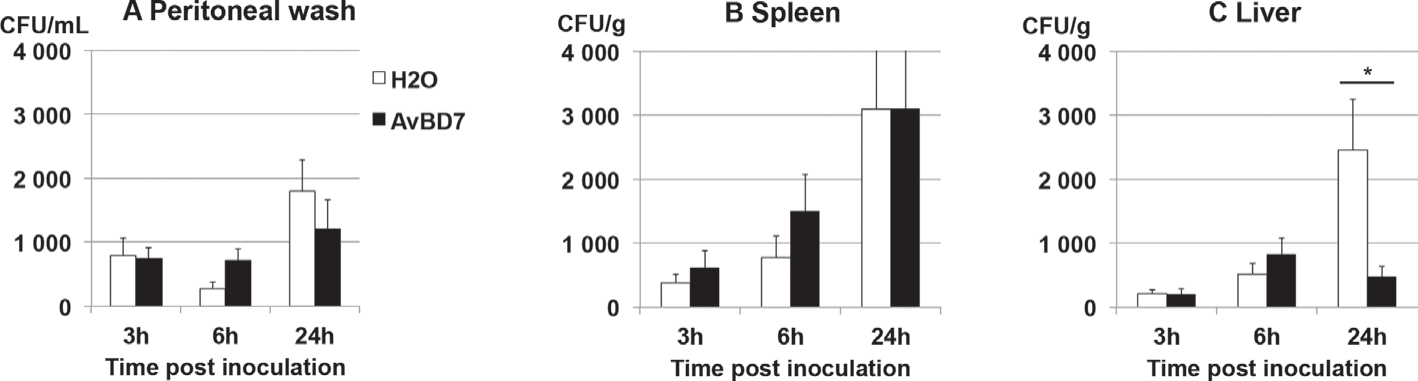
Kinetics of *Salmonella* multiplication in the peritoneal cavity or organs of infected mice after treatment or not with AvBD7. BALB/c mice infected by *S*. Typhimurium were treated with AvBD7 (black bars) or not (white bars), as indicated in the Materials and Methods. Values are given as mean number of CFU per ml of peritoneal liquid **(A)** or per g of spleen **(B)** or liver **(C)**, at the indicated time in h, ± standard error of the mean (*n* = 10). Asterisk indicates significant difference between treated and mock treated mice (*p* = 0.03).

The effect of AvBD7 repeated treatment on the bacterial load was investigated 3, 6, and 24 h p.i. either in the peritoneal cavity or in the organs of the reticulo-endothelial system, as shown in Figure 6. The bacterial load in the peritoneal cavity (Figure 6A) reached 1.8 × 10^3^ CFU/ml at 24 h p.i. This was indicative of a local multiplication of *S.* Typhimurium DT104 during the first 24 h p.i., which was unaffected by AvBD7 treatment, even if the bacterial load was slightly lower at 24 h p.i. in the treated group in comparison to the control group. Bacterial colonization in organs of the reticulo-endothelial system was confirmed by the measurement of about 3 × 10^3^ CFU/g in the spleen and of about 2.5 × 10^3^ CFU/g in the liver of non-treated mice at 24 h p.i. (Figures 6B,C, respectively). There was no significant effect of AvBD7 repeated administration on the splenic bacterial load over time (Figure 6B). By contrast, AvBD7 showed a significant inhibitory effect on bacterial colonization of the liver, with 80% reduction in the number of bacteria observed at 24 h p.i. in the treated group in comparison to the mock-treated group (*p =* 0.03) (Figure 6C). Bacterial load significantly increased over time in the liver of infected and non-treated mice while it was maintained at low level in AvBD7-treated mice.

#### Effect on Mice Survival

**Figure 7:**
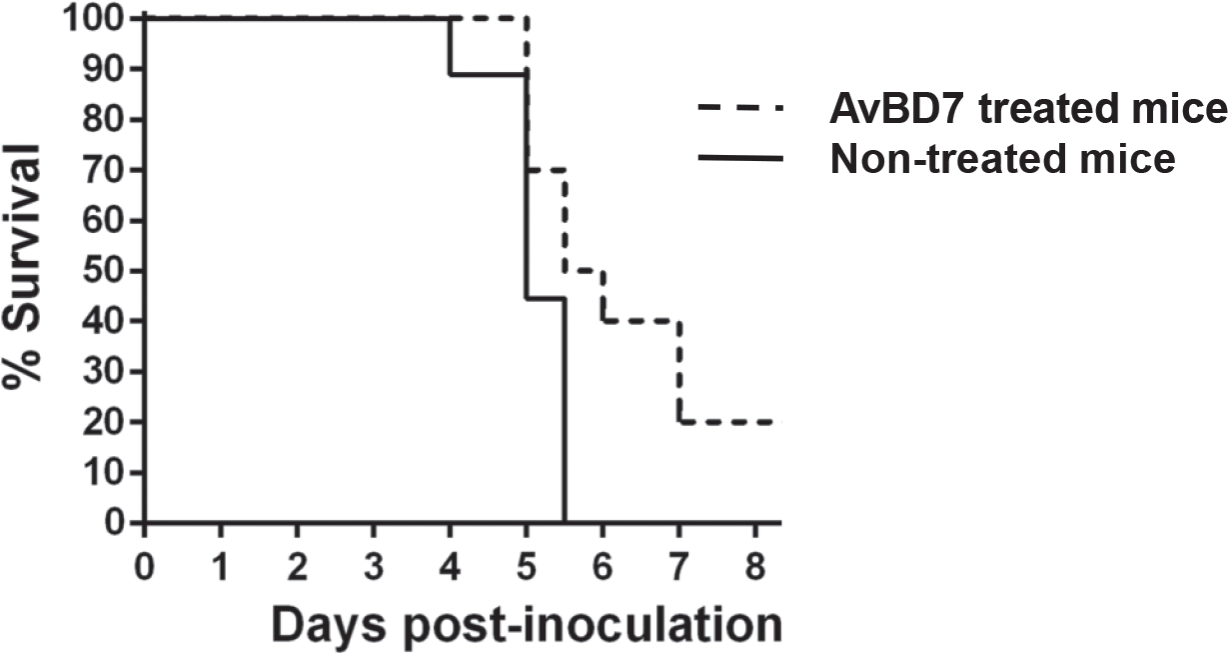
Kinetics of mice survival after inoculation of *S*. Typhimurium and two injections of 100 μg of AvBD7. BALB/c mice were inoculated by the intraperitoneal route with 140 CFU of *S.* Typhimurium DT104 strain, and were injected twice over 2 h interval by the same route with 100 μl sterile endotoxin-free H_2_O containing 100 μg AvBD7 (200 μg total per treated animal; dotted line) or not (untreated; full line). Log-rank test indicated significant differences between treated (*n* = 10) and mock-treated (*n* = 10) group of mice (*p* = 0.02).

Finally, the effect of AvBD7 treatment on the survival of *Salmonella* infected mice was investigated, as shown in Figure 7. Animals infected and non-treated started to die 4 days after inoculation. They reached 100% mortality before 6 days of infection. By contrast, two injections of AvBD7 (10 mg/kg of body weight) led to a significant delay in mortality with 50% of animals surviving until day 6 p.i. as compared to 0% in the non-treated control group (*p* = 0.03). Finally, there was still 20% survival in the treated group while 0% survival in the non-treated group after 1 week of infection. The overall survival curves between groups were significantly different (*p* = 0.02).

## Discussion

The antibacterial effect of AvBD7 on MDR *S.* Typhimurium strain attested by a MIC below 4 μM (or 20 mg/l) prompted us to investigate the therapeutic efficacy of AvBD7 against this strain able to induce 100% mortality in mice after 7 days of infection with 140 CFU given by intraperitoneal route. This model is particularly severe in mice due to extremely low lethal dose of *S*. Typhimurium. Two i.p. administrations of AvBD7 at 10 mg/kg showed beneficial effects by increasing mice survival in the model of systemic lethal salmonellosis, consistently correlated with the reduction of bacterial load in the liver of treated animals. Maiti and colleagues have shown a higher beneficial effect of human defensins HBD1 and HBD2 on mice survival in a murine model of salmonellosis using *S*. Typhi as infectious agent (Maiti et al., 2014). This discrepancy could be due to a better effect of these human defensins in the homologous infectious model.

The ability of AvBD7 to reduce bacterial load in the liver of *Salmonella-*infected animals is in agreement with the distribution of this defensin to this organ. Indeed, even if most part of the fluorescence-labeled AvBD7 injected in the peritoneal cavity of mice reached bloodstream, kidneys and was eliminated through the urinary tract, a substantial amount was found in the liver, the second most fluorescent organ after the kidneys. A similar study performed in mice with a proline-rich peptide named Bac7 has also revealed the very fast renal excretion rate of the antimicrobial peptide (Benincasa et al., 2010). This peptide injected in the peritoneal cavity at 30 mg/kg in *S*. Typhimurium-infected mice exhibited also beneficial effect on mice survival, without significant reduction of the liver bacterial load. However, this peptide is shorter and probably less structured than AvBD7, being theoretically more susceptible to proteolysis, which may explain why it was eliminated within 5 h following i.p. injection. In the present study, AvBD7 was still present in deep organs at 24 h postinjection, highlighting its stability. Therefore, AvBD7 may circulate to the liver in the bloodstream. Elsewhere, AvBD7 was shown associated to peritoneal cells whose macrophages constitute the major cell population. Beta-defensin HBD3 entry inside macrophages has been documented by Semple and colleagues (Semple et al., 2011; Semple and Dorin, 2012). Since AvBD7 was also shown to enter macrophages, it may circulate under an intracellular form inside macrophages by a phenomenon called “macrophages disappearance reaction” corresponding to macrophage migration toward the liver, which is induced by i.p. challenge with bacterial components (Barth et al., 1995). Moreover, recent data have interestingly suggested the peritoneal cavity as a reservoir for macrophage migration, *via* a non-vascular route, to injured liver under inflammatory conditions (Wang and Kubes, 2016). Macrophages may thus serve as vehicle for AvBD7 dissemination to the liver, which could explain its beneficial antibacterial effects in this organ following systemic administration.

Keeping in mind that *Salmonella* displays a preferential tropism for macrophages, both for survival and dissemination purposes (Mastroeni and Grant, 2011; Ruby et al., 2012; Behnsen et al., 2015), it is tempting to hypothesize that AvBD7 may exert an effect on bacterial clearance through a direct or indirect (stimulating) antibacterial activity inside macrophages. The non-cytotoxic effect of AvBD7 coupled to its capacity to enter macrophages and to reduce intracellular bacterial load corroborate such mechanism. A similar intracellular antibacterial effect in macrophages for the human cathelicidin LL37 also supports this mechanism of cell penetrating active peptide (Tang et al., 2015). In the present study, strategies to reduce either membrane fluidity, such as incubations at 4°C and with nystatin, or cytoskeleton rearrangement such as cytochalasin D both inhibited AvBD7 internalization. It was however unaffected by blocking coated pits formation by clathrin using chlorpromazine as inhibitor. AvBD7 thus appears internalized through an endocytosis-like mechanism, which may allow it to reach and kill bacteria inside the macrophage. Future work will aim at investigating the involvement of a hypothetical receptor in the ability of AvBD7 to display antibacterial activity inside infected cells and to stimulate host cells for immune modulatory activity.

In conclusion, an anti-*Salmonella* effect of AvBD7 demonstrated in infected macrophages is consistent with its systemic beneficial effect on the reduction of liver bacterial load and on the survival of mice when applied after challenge with a MDR *Salmonella* strain. AvBD7 thus appears as an interesting candidate as alternative or complementary to conventional antibiotics in the treatment of bacterial infections of major concern for veterinary medicine and public health.

## Author Contributions

GB and RG acquired, analyzed, interpreted data, and designed the study. A-CL obtained funding, analyzed and interpreted data, and designed all study. IV-P, JT, and CS acquired, analyzed, and interpreted the data, and designed animal infection study. IL acquired, analyzed, and interpreted data and designed the animal imaging study. PV and CS obtained funding. A-CL drafted the manuscript. FG, AW, and AT technically supported and interpreted data for defensin preparation, confocal microscopy, and animal infection study, respectively. All authors reviewed the final manuscript.

### Conflict of Interest Statement

The authors declare that the research was conducted in the absence of any commercial or financial relationships that could be construed as a potential conflict of interest.

## Footnotes

^1^http://aps.unmc.edu/AP/main.php

^2^https://www.efsa.europa.eu/fr/efsajournal/pub/4634
